# Genome Sequences of Multidrug-Resistant *Acinetobacter baumannii* Strains from Nosocomial Outbreaks in Japan

**DOI:** 10.1128/genomeA.00476-13

**Published:** 2013-07-18

**Authors:** Masato Suzuki, Mari Matsui, Satowa Suzuki, Emiko Rimbara, Satomi Asai, Hayato Miyachi, Tohru Takata, Yoichi Hiraki, Fumio Kawano, Keigo Shibayama

**Affiliations:** Department of Bacteriology II, National Institute of Infectious Diseases, Musashimurayama, Tokyo, Japana; Department of Laboratory Medicine, Tokai University School of Medicine, Isehara, Kanagawa, Japanb; Department of Infection Control, Fukuoka University Hospital, Nanakuma, Jonan-ku, Fukuoka, Japanc; Department of Pharmacy National Hospital, Organization Kumamoto Medical Center, Ninomaru, Chuo-ku, Kumamoto, Japand; Department of Hematology, National Hospital, Organization Kumamoto Medical Center, Ninomaru, Chuo-ku, Kumamoto, Japane

## Abstract

*Acinetobacter baumannii* has emerged worldwide as an important nosocomial pathogen in medical institutions. Here, we present the draft genome sequences of *A. baumannii* strains MRY09-0642, MRY10-0558, and MRY12-0277 that were isolated from nosocomial outbreaks in Japan between 2008 and 2012 and that are resistant to antimicrobial agents, including carbapenems, fluoroquinolones, and aminoglycosides.

## GENOME ANNOUNCEMENT

*Acinetobacter baumannii* often causes infections in hospitalized immunocompromised patients (1). *A. baumannii* strains belonging to international clone II (IC2)/sequence type 2 (ST2), the most prevalent epidemic lineage, are associated with multidrug resistance and often cause nosocomial outbreaks (2). Since 2000, carbapenem-resistant and multidrug-resistant *A. baumannii* strains have emerged and their prevalence has increased worldwide. In some countries, carbapenem-resistant strains have reached a prevalence of >50%, becoming a serious public health threat (3). According to national surveillance data from Japan Nosocomial Infections Surveillance (JANIS) conducted by the Ministry of Health, Labour and Welfare (http://www.nih-janis.jp/english/), carbapenem resistance among *Acinetobacter* spp. in Japan remains much lower than in other countries (approximately 2% of imipenem resistance in Japan in 2012) (S. Suzuki, unpublished data). However, the prevalence tends to increase gradually, and nosocomial outbreaks of *A. baumannii* IC2 infections have occasionally occurred in Japan.

To date, whole-genome sequences of *A. baumannii* strains isolated in Japan have not been available in GenBank. In this report, we announce the availability of the draft genome sequences of *A. baumannii* MRY09-0642, MRY10-0558, and MRY12-0277, which caused nosocomial outbreaks in geographically different medical institutions in Japan in 2008, 2010, and 2012, respectively. These isolates were classified as IC2 by multilocus sequence typing (4), whereas they showed distinct ApaI fragment patterns in pulsed-field gel electrophoresis. Whole-genome shotgun (WGS) sequencing of the *A. baumannii* strains was performed using the Roche 454 pyrosequencing platform (500-bp insert size). Reads were assembled with Newbler assembler version 2.3 (Roche), using *A. baumannii* Taiwanese strain MDR-TJ (5) as the reference.

The draft genome sequences of *A. baumannii* MRY09-0642, MRY10-0558, and MRY12-0277 consist of 147, 77, and 106 contigs, respectively, yielding total sequences for each strain of 3,746,543, 3,782,742, and 3,829,745 bp, with *N*_50_ contig sizes of 64,650, 164,570, and 91,207 bp, respectively. Their mean G+C content is 39.0% ± 0.1%. A total of 3,645, 3,604, and 3,735 coding genes for MRY09-0642, MRY10-0558, and MRY12-0277, respectively, were detected by the RAST server (http://rast.nmpdr.org) (6). Acquired antimicrobial resistance genes in the WGS data were identified using a Web-based tool, ResFinder version 1.3 (http://cge.cbs.dtu.dk/services/ResFinder/) (7). *A. baumannii* MRY09-0642, MRY10-0558, and MRY12-0277 carry OXA-51-like β-lactamase genes, which predominantly confer carbapenem resistance in *A. baumannii* (8), found as *bla*_OXA-82_ in contig 00088, *bla*_OXA-254_ in contig 00034, and *bla*_OXA-66_ in contig 00056, respectively.

A more-detailed report of the drug resistance and virulence phenotypes of these three *A. baumannii* strains will be included in a future publication. Access to these genome sequences and their comparative analyses with other epidemic and nonepidemic strains will facilitate additional comprehensive bioinformatics and phylogenetic analyses, thus expanding our understanding of the global public health problem caused by this nosocomial pathogen.

### Nucleotide sequence accession numbers.

These WGS projects have been deposited at DDBJ/EMBL/GenBank under the accession no. BASA00000000, BASB00000000, and BASC00000000. The versions described in this report are the first versions, accession no. BASA01000000, BASB01000000, and BASC01000000 for *A. baumannii* strains MRY09-0642, MRY10-0558, and MRY12-0277, respectively. 
